# An Adaptive Iterative Learning Based Impedance Control for Robot-Aided Upper-Limb Passive Rehabilitation

**DOI:** 10.3389/frobt.2019.00041

**Published:** 2019-06-04

**Authors:** Wang Ting, Song Aiguo

**Affiliations:** School of Instrument Science and Engineering, Southeast University, Nanjing, China

**Keywords:** impedance control, anthropomorphic arm, pneumatic artificial muscles, iterative learning, rehabilitation

## Abstract

In this paper, an anthropomorphic arm is introduced and used to the upper-limb passive rehabilitation therapy. The anthropomorphic arm is constructed via pneumatic artificial muscles so that it may assist patients suffering upper-limb diseases to achieve mild therapeutic exercises. Due to the uncertain dynamic environment, external disturbances and model uncertainties, a combined control is proposed to stabilize and to enhance the adaptivity of the system. In the combined control, an iterative learning control is used to realize accurate position tracking. Meanwhile, an adaptive iterative learning based impedance control is proposed to execute the appropriate contact force during the therapy of the upper-limb. The advantage of the combined control is that it doesn't depend on the accurate model of systems and it may deal with highly nonlinear system which has strong coupling and redundancies. The convergence of the proposed control is analyzed in detail. Numerical simulations are performed to verify the proposed control method. In addition, real experiments are executed on the Southwest anthropomorphic arm.

## 1. Introduction

China ranks the first in the incidence of the stroke in the world, accounting for one third of the world's 30 million stroke patients. According to the report on cardiovascular diseases in China 2018, at present, there are more than 13 million stroke patients in China (Shengshou et al., 2019). If further rehabilitation measures are not taken, there will be 31 million stroke patients all over the country till 2030. About 75% of the stroke survivors have different degrees of disabilities and most of stroke patients are unable to take care of themselves. Therefore, stroke patients need to execute rehabilitation exercises in order to adapt to activities of daily living (ADL) by themselves (Morales et al., 2011). In early time, many upper-limb therapies are developed to help stroke patients recovering their motion skills, such as constraint-induced movement therapy (Taub and Uswatte, 2006), bilateral training (Tijs and Matyas, 2006) and so on. However, these therapies always rely on the skills and experiences of physiotherapists, which are usually expensive and time consuming. In recent years, robot-aided therapies for upper-limb rehabilitation are rising up due to the low price and the high efficiency (Papageorgiou et al., 2006; Ball et al., 2007, 2009; Mehdi, 2012).

Comparing with electric drives, pneumatic actuators have advantages of the light weight, the high strength, the compliance, and the low impedance. Specially, they have a high power-to-weight ratio, the low inherent impedance, and forces are controllable (Maciejasz et al., 2014). Therefore, researchers are interested in introducing the pneumatic actuators to robot-aided upper-limb rehabilitation therapies. The university of Leeds designs the iPam system for sitting therapies, consisting of two symmetric arms and each arm has 3 DOF (Degrees of freedom) (Jackson A. E. et al., 2007). An admittance control is used to achieve patients' arm movements. The system is equipped with force sensors, a motion capture software and infrared cameras. To prevent suffering serious damage, a cooperative control is integrated to make positions of two robotic arms restricted to the kinematics of the human arm (Jackson A. et al., 2007). The university of California develops a PNEU-WREX system, using pneumatic actuators for upper-limb rehabilitation therapies. The system has 5 DOF, immersing in a virtual environment (Wolbrecht et al., 2010). Applied pneumatic cylinders, every active DOF uses corresponding valves to implement the low pressure control loop so as to exert the Kalman filter and the force control for the nonlinear system. Low-friction cylinders are used to solve the friction among pneumatic cylinders and the system is performed by a passive gravity compensation of the patients' arm weights. The system is equipped with pressure sensors, cameras, MEMs interferometers and the XPC type data acquisition card (DAQ). Some results demonstrate that the T-WREX may attenuate moderate to severe upper extremity hemiparesis of stroke patients through repetitive motor training (Housman et al., 2007). The University of Salford fabricates a 7 DOF multi-joint gravity compensated upper-limb exoskeleton device, called Salford rehabilitation exoskeleton (SRE). The device takes pneumatic muscles to emulate agonist-antagonist muscles of the human arm. Every joint has three mode of operations, totally assisted mode, partial assistance mode and none assistance mode, all of which are accomplished by the position control, the torque control or the impedance control. The device is used to assist rehabilitation exercises of the patients' upper-limb (Kousidou et al., 2007).

In the robot-aided upper-limb rehabilitation exercises, the safety is the most important issue for both the rehabilitation device and patients. Therefore, the accomplishment of the compliant control system is the key point in the rehabilitation robot design. The impedance control is the simple and efficient approach to provide the safe and compliant contact force, via adjusting the dynamic relation between robot's end-effector and the patients (Kiguchi et al., 2003; Xu and Fang, 2004; Ju et al., 2005; Kooij et al., 2006; Nakamura et al., 2008; Ball et al., 2009; Kang et al., 2010; Xu and Song, 2010; Mehdi, 2012). During the motion, using constant parameters may result ineffectiveness of the impedance control since the impedance model parameters of the environment are time-varying. To solve the problem, Xu and his colleagues study the force-position hybrid fuzzy control of a rehabilitation robot with uncertain dynamic system (Xu and Fang, 2004). kiguchi and his colleagues combine the fuzzy logic with the neural network to the proposed impedance controller in order to solve the nonlinearities and uncertainties of the system (Kiguchi et al., 2003). Even more, some researchers propose an adaptive impedance control algorithm based on Dynamic Recurrent Fuzzy Neural Network (DRFNN) aiming to execute rehabilitation program more effectively. Impedance model parameters of impaired limb's are identified in real time, and desired impedance control parameters are learned by the DRFNN at the same time. The effectiveness of the method is verified by simulation experiments (Xu and Song, 2010). Due to highly nonlinear and time varying characteristics, it is difficult to drive PAM actuators in the precise control. Therefore, Zhang and his colleagues investigate the force control of a pneumatic artificial muscle (PAM) drives ankle rehabilitation robot, incorporating a position control in inner loop and an impedance control in outer loop so as to ensure the accuracy and the satisfactory of the ankle rehabilitation (Zhang et al., 2017). Anh and his colleagues develop the novel adaptive neural network (ADNN) compliant force/position control to make a serial PAM robot follow arbitrary linear and circular trajectories. Through experiments and the comparison with optimal PID control, they prove that the proposed method may improve the compliant force/position output performance (Anh et al., 2017). Although researchers already study various methods, it is still a tough problem and an active topic how to make a balance between the accurate motion control and the appropriate contact force during rehabilitation therapies.

In this paper, we firstly introduce the 2 DOF pneumatic drives anthropomorphic arm in our lab. The anthropomorphic arm forms by 6 PAMs, and equipped with 3 dimension force sensor and other sensors. To realize the accurate motion control, an iterative learning control (ILC) is applied to make the anthropomorphic arm follow arbitrary trajectories. Simultaneously, an adaptive iterative learning based impedance force control is proposed to maintain the stability of the anthropomorphic arm and to acquire the appropriate contact force during the motion. Rest of the paper is organized as follows. The pneumatic anthropomorphic arm and its dynamic model are presented in section 2. The adaptive iterative learning based impedance force control is detailed explained and analyzed in section 3. Results of numerical simulations and real experiments are discussed in section 4. Conclusion and the future work are given in the conclusion part.

## 2. Pneumatic Drives Anthropomorphic Arm and Its Dynamic Equation

**Figure 1:**
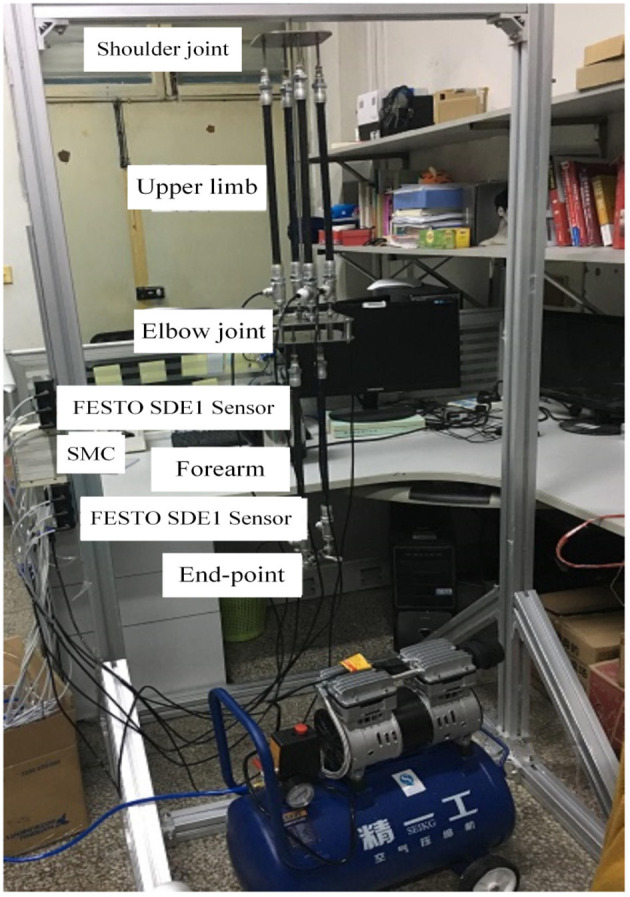
The name of anthropomorphic arm is Southwest, located in the School of Instrument Science and Engineering, Southeast University, China.

**Figure 2:**
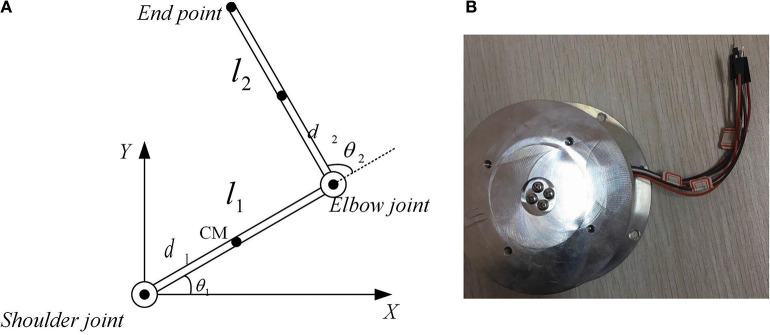
The anthropomorphic arm model and the 3 dimension force sensor in State Key Laboratory of Bioelectronics, Jiangsu Key Lab of Remote Measurement and Control, School of Instrument Science and Engineering, Southeast University, 210096 P.R.China. **(A)** The anthropomorphic arm model, **(B)** 3 dimension force sensor.

The anthropomorphic arm (called Southwest) in the State Key Laboratory of Bioelectronics has the shoulder joint, the elbow joint and the end-effector, as shown in Figure 1. Both of the elbow joint and the end-effector has 1 rotational DOF and the Southwest together has 2 rotational DOF. All of joints are realized by roundels embedded with cardan valves and connected with PAMs. The upper-limb is actuated by the muscle synergies of 4 PAMs and the forearm is actuated by another 2 PAMs. In the upper-limb, 4 PAMs form 2 pairs agonist-antagonist muscles as similar as the human arm. The end-effector is equipped with the 3 dimension force sensor (designed and manufactured by State Key Laboratory of Bioelectronics, and showed in Figure 2A) and the IMU sensor, used to measure the forces, positions, angles and angular velocities. Every pneumatic artificial muscle may execute the extension or the flexion via inflating or deflating gases of corresponding SMC pneumatic proportional valve. FESTO SDE1 pneumatic pressure sensors are equipped to measure increments of gases. Through the synergism of toques generated by all PAMs, the end-effector of the anthropomorphic arm may achieve mild reaching movements.

The kinematic model of the human arm is illustrated in Figure 2, and the dynamic equation of the Southwest anthropomorphic arm is written as

(1) $$\begin{array}{l} M\left(q\right)\ddot{q}+C\left(q,\dot{q}\right)\dot{q}+G\left(q\right)=\tau -\tau_{d}. \end{array}$$

In Equation (1), *M* represents the inertia term. *C* indicates the Coriolis and centrifugal effects term. *G*(*q*) is the gravitational item. τ is the joint torque generated by the synergism of PAMs acting on the elbow joint and the end-effector. τ_*d*_ represents disturbances and perturbations. $q={\left[q_{1},q_{2}\right]}^{T}={\left[\theta_{1},\theta_{2}\right]}^{T}$, $\tau ={\left[\tau_{1},\tau_{2}\right]}^{T}$. The specific forms of *M* and *C* are, respectively defined as:

(2) $$\begin{array}{lll} M & = & \left[\begin{array}{cc} J_{1}+J_{2}+m_{2}d_{1}^{2}+2m_{2}d_{1}c_{2}\cos q_{2} & J_{2}+m_{2}d_{1}c_{2}\cos q_{2}\\ J_{2}+m_{2}d_{1}c_{2}\cos q_{2} & J_{2} \end{array}\right],\\ C & = & \left[\begin{array}{cc} -2m_{2}d_{1}c_{2}{\dot{q}}_{2}\sin q_{2} & -m_{2}d_{1}c_{2}\sin q_{2}\left({\dot{q}}_{1}+{\dot{q}}_{2}\right)\\ m_{2}d_{1}c_{2}{\dot{q}}_{1}\sin q_{2} & 0 \end{array}\right],\\ G & = & \left[\begin{array}{c} g\left(m_{1}c_{1}+m_{2}d_{1}\right)\cos q_{1}+gm_{2}c_{2}\cos\left(q_{1}+q_{2}\right)\\ gm_{2}c_{2}\cos\left(q_{1}+q_{2}\right) \end{array}\right]. \end{array}$$

In Equation (2b), *m*_1_ and *m*_2_ are masses of the upper-limb and the forearm. *g* is the gravitational coefficient. *d*_1_ and *d*_2_ are lengths of the upper-limb and the forearm. *d*_*i*_ is the distance from the i-th joint to the center of mass of the i-th link. *I*_*i*_ represents the inertia moment of the i-th link, and the inertia matrix *J*_*i*_ calculates as $J_{i}=m_{i}d_{i}^{2}+I_{i}$.

**Figure 3:**
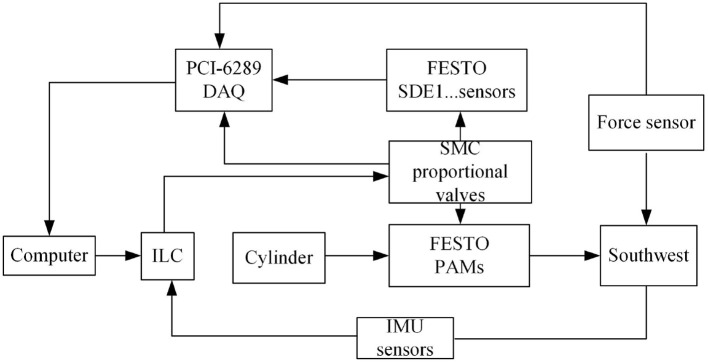
The platform of the robot-aided upper-limb rehabilitation system.

The schematic diagram of the robot-aided upper-limb rehabilitation system is displayed in Figure 3. All of SMC proportional valves, pneumatic sensors and force sensors are connected with PCI 6289 data acquisition cards (DAQs). The control executes by the labView written in an external computer. The Southwest adopts pure pneumatic drives supplied by the air source. The IMU sensors may obtain the actual positions, angles, and angular velocities of the end-effector and the elbow. Once the desired angle is given, the Southwest may follow the desired trajectory through the motion control. Assuming each PAM has the same contraction rate, gas variations of inflation or deflation have the following relation of the contraction rate ζ and forces *F*_*i*_.

(3) $$\begin{array}{l} F_{i}=-296+2000P_{i}\zeta {|}_{4bar,\zeta =0.08},i=1,...,6,\\ \tau_{1}=\sum_{i=1}^{4}F_{i}l_{i},\\ \tau_{2}=\sum_{i=5}^{6}F_{i}l_{i}, \end{array}$$

where *P*_*i*_ is the air pressure amount. *l*_*i*_ represents the arm of i-th PAM, *l*_*i*_ = 0.45*m*. Forces of antagonist muscles are both set to 0. Agonist muscles are supposed to have the same amounts of initial pressures. Therefore, the problem is simplified to calculate gas variations of agonist muscles via the control, depending on the position tracking errors between actual angles and desired angles. The classical PID based iterative learning control is used to implement the motion control, and it is omitted.

## 3. Adaptive Iterative Learning Based Impedance Control

In the robot-aided upper-limb rehabilitation training process, it is essential to design the contact force between the end-effector of the anthropomorphic arm and the upper-limb of the patient. If the interaction force is not properly controlled, it is not only fail to achieve the training effect, but also may lead to secondary injury of the patient's part. In actual rehabilitation exercises, it is very important to select appropriate parameters of the environment's impedance model. Generally, the parameters may be acquired by adaptive methods, or the neural network learning methods or from teaching data. In this paper, we propose to use iterative learning to obtain the parameters. The target impedance model used in this paper is expressed as

(4) $$\begin{array}{l} M_{d}{\ddot{q}}_{k}\left(t\right)+C_{d}{\dot{q}}_{k}\left(t\right)+S_{d}q_{k}\left(t\right)=F_{dk}\left(t\right)-F_{k}\left(t\right), \end{array}$$

where k indicates the number of the circulation period and $q_{k}\left(t\right),{\dot{q}}_{k}\left(t\right),{\ddot{q}}_{k}\left(t\right)$ are actual joint angles, angular velocities and accelerations of the Southwest in the k-th circulation period. Relatively, $q_{d}\left(t\right),{\dot{q}}_{d}\left(t\right),{\ddot{q}}_{d}\left(t\right)$ are the desired position vector, the desired velocity vector and the desired acceleration vector of the desired motion. *M*_*d*_, *C*_*d*_ and *S*_*d*_ are the variable matrix parameters of the environment's impedance model. *F*_*k*_(*t*) is the actual contact force of the Southwest measured by the 3 dimension force sensor. *F*_*dk*_(*t*) is the force vector imposed by the time-varying environment.

Assume that parameters of Equation (4) are unknown and the impedance model Equation (4) satisfies the following assumptions

1. For ∀*t* ∈ [0, *T*], *q*_*d*_(*t*), ${\dot{q}}_{t}$, ${\ddot{q}}_{t}$ and *F*_*dk*_(*t*) are all bounded.
2. The initial value satisfies ${\dot{q}}_{d}\left(0\right)-{\dot{q}}_{k}\left(t\right)=q_{d}\left(0\right)-q_{k}\left(0\right)=0$.
3. *M*_*d*_(*q*_*k*_) ∈ **R**_*n*×*n*_ is a symmetric positive definite matrix and is bounded.
4. Ṁ_*d*_ − 2*C*_*d*_ is a symmetric matrix and $x^{T}\left({Ṁ}_{d}-2C_{d}\right)x=0,\forall x\in {\text{R}}^{n}$.
5. $S_{d}q_{d}\left(t\right)+C_{d}{\dot{q}}_{d}\left(t\right)=\psi \left(q_{k},{\dot{q}}_{k}\right)\xi^{T}\left(t\right)$, where $\psi \left(q_{k},{\dot{q}}_{k}\right)\in {\text{R}}^{n\times \left(m-1\right)}$ is a given matrix and ξ^*T*^(*t*) ∈ **R**^*m*−1^ is an unknown vector.
6. $||C_{d}\left(q_{k},{\dot{q}}_{k}\right)||\leq k_{c}||{\dot{q}}_{k}||$, ||*S*_*d*_*q*_*k*_|| ≤ *k*_*g*_||*q*_*k*_||, ∀*t* ∈ [0, *T*], where *k*_*c*_ and *k*_*g*_ are positive real numbers.

If the impedance control law is designed as

(5) $$\begin{array}{l} \tau_{k}\left(t\right)=K_{P}{\tilde{q}}_{k}\left(t\right)+K_{D}{\dot{\tilde{q}}}_{k}\left(t\right)+\varphi \left(q_{k},{\dot{q}}_{k},{\dot{\tilde{q}}}_{k}\left(t\right)\right){\hat{\theta }}_{k}\\ {\hat{\theta }}_{k}\left(t\right)=\Gamma \varphi^{T}\left(q_{k},{\dot{q}}_{k},{\dot{\tilde{q}}}_{k}\left(t\right)\right){\dot{\tilde{q}}}_{k}\left(t\right), \end{array}$$

then ${\tilde{q}}_{k}\left(t\right)$, ${\dot{\tilde{q}}}_{k}\left(t\right)$ are both bounded, and $\underset{k\to \;\infty }{\lim}\;{\tilde{q}}_{k}(t)=\underset{k\to \;\infty }{\lim}\dot{\tilde{q}}(t)=0,\;\forall t\text{ ε [0, T]}$. In Equation (5), ${\hat{\theta }}_{-1}\left(t\right)=0$, ${\tilde{q}}_{k}\left(t\right)=q_{d}\left(t\right)-q_{k}\left(t\right)$, ${\dot{\tilde{q}}}_{k}\left(t\right)={\dot{q}}_{d}\left(t\right)-{\dot{q}}_{k}\left(t\right)$, $\varphi \left(q_{k},{\dot{q}}_{k},{\dot{\tilde{q}}}_{k}\left(t\right)\right)\in {\text{R}}^{n\times n}$, and $\varphi^{T}\left(q_{k},{\dot{q}}_{k},{\dot{\tilde{q}}}_{k}\left(t\right)\right)\overset{def}{=}\left[\text{Ψ}\left(q_{k},{\dot{q}}_{k}\right)sgn\left({\dot{\tilde{q}}}_{k}\right)\right]$. Matrices $K_{p}\in {\text{R}}^{n\times n}$, $K_{D}\in {\text{R}}^{n\times n}$ and Γ ∈ **R**^*m*×*m*^ are positive definite symmetric matrices.

The the convergence proof of the proposed impedance control is explained as follows.

a. The boundedness proof of Δ*W*_*k*_.

The Lyapunov function is designed as

(6) $$\begin{array}{l} W_{k}\left({\dot{\tilde{q}}}_{k}\left(t\right),{\tilde{q}}_{k}\left(t\right),{\tilde{\theta }}_{k}\left(t\right)\right)=V_{k}\left({\dot{q}}_{t},{\tilde{q}}_{k}\left(t\right)\right)+\frac{1}{2}\int_{0}^{t}{\tilde{\theta }}_{k}\left(t\right)\Gamma^{-1}{\tilde{\theta }}_{k}\left(t\right)d\tau , \end{array}$$

where ${\tilde{\theta }}_{k}\left(t\right)=\theta \left(t\right)-{\hat{\theta }}_{k}\left(t\right)$, θ(*t*) = [ξ^*T*^(*t*), β]^*T*^. ${\hat{\theta }}_{k}\left(t\right)={\left[{\hat{\xi }}_{k}^{T}\left(t\right),{\hat{\beta }}_{k}\left(t\right)\right]}^{T}$ is the estimated value of θ(*t*) and $||M_{d}{\ddot{q}}_{d}-F_{dk}\left(t\right)||\leq \beta$. The $V_{k}\left({\dot{\tilde{q}}}_{t},{\tilde{q}}_{k}\left(t\right)\right)$ is set as

(7) $$\begin{array}{l} V_{k}\left({\dot{\tilde{q}}}_{k}\left(t\right),{\tilde{q}}_{k}\left(t\right)\right)=\frac{1}{2}{\dot{\tilde{q}}}_{k}^{T}M_{d}{\dot{\tilde{q}}}_{k}+\frac{1}{2}{\tilde{q}}_{k}^{T}K_{P}{\tilde{q}}_{k}. \end{array}$$

Due to ${\bar{\theta }}_{k}=-\theta +{\hat{\theta }}_{k}-{\tilde{\theta }}_{k-1}+\theta ={\tilde{\theta }}_{k-1}-{\tilde{\theta }}_{k}$, we have ${\tilde{\theta }}_{k-1}={\bar{\theta }}_{k}+{\tilde{\theta }}_{k}$ and

$$\begin{array}{l} {\tilde{\theta }}_{k}^{T}\Gamma^{-1}{\tilde{\theta }}_{k}-{\tilde{\theta }}_{k-1}^{T}\Gamma^{-1}{\tilde{\theta }}_{k-1}={\tilde{\theta }}_{k}^{T}\Gamma^{-1}{\tilde{\theta }}_{k}-\left({\bar{\theta }}_{k}+{\tilde{\theta }}_{k}^{T}\right){\tilde{\theta }}_{k-1}^{T}\Gamma^{-1}\left({\bar{\theta }}_{k}+{\tilde{\theta }}_{k}\right)\\ \;=-{\bar{\theta }}_{k}^{T}\Gamma^{-1}{\bar{\theta }}_{k}-{\bar{\theta }}_{k}^{T}\Gamma^{-1}{\tilde{\theta }}_{k}-{\tilde{\theta }}_{k}^{T}\Gamma^{-1}{\bar{\theta }}_{k}=-{\bar{\theta }}_{k}^{T}\Gamma^{-1}{\bar{\theta }}_{k}-2{\bar{\theta }}_{k}^{T}\Gamma^{-1}{\tilde{\theta }}_{k}. \end{array}$$

Then

(8) $$\begin{array}{l} \Delta W_{k}=W_{k}-W_{k-1}=V_{k}-V_{k-1}\\ \;+\frac{1}{2}\int_{0}^{t}\left({\tilde{\theta }}_{k}^{T}\Gamma^{-1}{\tilde{\theta }}_{k}-{\tilde{\theta }}_{k-1}^{T}\Gamma^{-1}{\tilde{\theta }}_{k-1}\right)d\tau \\ \;=V_{k}-V_{k-1}-\frac{1}{2}\int_{0}^{t}\left({\bar{\theta }}_{k}^{T}\Gamma^{-1}{\bar{\theta }}_{k}^{T}+2{\bar{\theta }}_{k}^{T}\Gamma^{-1}{\tilde{\theta }}_{k}\right)d\tau . \end{array}$$

Due to $\int_{0}^{t}{\dot{V}}_{k}\left(t\right)d\tau =V_{k}\left(t\right)-V_{k}\left(0\right)$, and ${\dot{V}}_{k}\left(t\right)=\frac{1}{2}{\dot{\tilde{q}}}_{k}^{T}M_{d}{\ddot{\tilde{q}}}_{k}+\frac{1}{2}{\dot{\tilde{q}}}_{k}^{T}{Ṁ}_{d}{\dot{\tilde{q}}}_{k}+{\dot{\tilde{q}}}_{k}^{T}K_{P}{\tilde{q}}_{k}$, we have

(9) $$\begin{array}{l} V_{k}\left({\dot{\tilde{q}}}_{k}\left(t\right),{\tilde{q}}_{k}\left(t\right)\right)=V_{k}\left({\dot{\tilde{q}}}_{k}\left(0\right),{\tilde{q}}_{k}\left(0\right)\right)\\ \;+\int_{0}^{t}\left({\dot{\tilde{q}}}_{k}^{T}M_{d}{\ddot{\tilde{q}}}_{k}+\frac{1}{2}{\dot{\tilde{q}}}_{k}^{T}{Ṁ}_{d}{\dot{\tilde{q}}}_{k}+{\dot{\tilde{q}}}_{k}^{T}K_{p}{\tilde{q}}_{k}\right)d\tau \end{array}$$

According to Equation (4) and assumptions, we have

$$\begin{array}{l} \;{\dot{\tilde{q}}}_{k}^{T}M_{d}{\ddot{\tilde{q}}}_{k}={\dot{\tilde{q}}}_{k}^{T}M_{d}\left({\ddot{q}}_{d}-{\ddot{q}}_{k}\right)={\dot{\tilde{q}}}_{k}^{T}M_{d}{\ddot{q}}_{d}\\ \;-{\dot{\tilde{q}}}_{k}^{T}\left(-C_{d}{\dot{q}}_{k}-S_{d}q_{k}+\tau_{k}+F_{dk}\right)\\ \;\frac{1}{2}{\dot{\tilde{q}}}_{k}^{T}{Ṁ}_{d}{\dot{\tilde{q}}}_{k}={\dot{\tilde{q}}}_{k}^{T}C_{d}{\dot{q}}_{d}-{\dot{\tilde{q}}}_{k}^{T}C_{d}{\dot{q}}_{k}\\ {\dot{\tilde{q}}}_{k}^{T}\left(M_{d}{\ddot{q}}_{d}-F_{dk}\right)\leq ||{\dot{\tilde{q}}}_{k}^{T}||\beta ={\dot{\tilde{q}}}_{k}^{T}\beta sgn\left({\dot{\tilde{q}}}_{k}\right)\\ \;\Psi \left(q_{k},{\dot{q}}_{k}\right)\xi^{T}+\beta sgn\left({\dot{\tilde{q}}}_{k}\right)=\left[\text{Ψ}\left(q_{k},{\dot{q}}_{k}\right),sgn\left({\dot{\tilde{q}}}_{k}\right)\right]\left[\xi^{T},\beta \right]\\ \;=\varphi \left(q_{k},{\dot{q}}_{k},{\dot{\tilde{q}}}_{k}\right)\theta . \end{array}$$

Then, we have

(10) $$\begin{array}{l} V_{k}({\dot{\tilde{q}}}_{k}(t),{\tilde{q}}_{k}(t))=V_{k}({\dot{\tilde{q}}}_{k}(0),{\tilde{q}}_{k}(0))+\int_{0}^{t}{\dot{\tilde{q}}}_{k}^{T}(M_{d}{\ddot{q}}_{d}-F_{dk}\\ \;+\;C_{d}{\dot{q}}_{d}+S_{d}q_{d}+K_{P}{\tilde{q}}_{k}-\tau_{k})\\ \;\leq V_{k}({\dot{\tilde{q}}}_{k}(0),{\tilde{q}}_{k}(0))+\int_{0}^{t}{\dot{\tilde{q}}}_{k}^{T}(\Psi (q_{k},{\dot{q}}_{k})\xi +K_{P}{\tilde{q}}_{k}+\beta sgn({\dot{\tilde{q}}}_{k})-\tau_{k})d\tau \\ \;\leq V_{k}({\dot{\tilde{q}}}_{k}(0),{\tilde{q}}_{k}(0))+\int_{0}^{t}{\dot{\tilde{q}}}_{k}^{T}(\varphi (q_{k},{\dot{q}}_{k},{\dot{\tilde{q}}}_{k})\theta +K_{P}{\tilde{q}}_{k}-\tau_{k})d\tau . \end{array}$$

Substituting ${\bar{\theta }}_{k}\left(t\right)={\left(\Gamma \varphi^{T}{\dot{\tilde{q}}}_{k}\right)}^{T}={\dot{\tilde{q}}}_{k}^{T}\varphi \Gamma$ and the control law into Equation (10), we have

(11) $$\begin{array}{l} V_{k}\left({\dot{\tilde{q}}}_{k}\left(t\right),{\tilde{q}}_{k}\left(t\right)\right)\leq V_{k}\left({\dot{\tilde{q}}}_{k}\left(0\right),{\tilde{q}}_{k}\left(0\right)\right)\\ \;+\int_{0}^{t}{\dot{\tilde{q}}}_{k}^{T}\left(\varphi \left(q_{k},{\dot{q}}_{k},{\dot{\tilde{q}}}_{k}\right){\tilde{\theta }}_{k}-K_{D}{\dot{\tilde{q}}}_{k}\right)d\tau , \end{array}$$

and

(12) $$\begin{array}{l} \;{\bar{\theta }}_{k}^{T}\Gamma^{-1}{\bar{\theta }}_{k}={\dot{\tilde{q}}}_{k}^{T}\varphi \Gamma \Gamma^{-1}\Gamma \varphi^{T}{\dot{\tilde{q}}}_{k}={\dot{\tilde{q}}}_{k}^{T}\varphi \Gamma \varphi^{T}{\dot{\tilde{q}}}_{k}\\ 2{\bar{\theta }}_{k}^{T}\Gamma^{-1}{\tilde{\theta }}_{k}=2{\dot{\tilde{q}}}_{k}^{T}\varphi \Gamma \Gamma^{-1}{\tilde{\theta }}_{k}=2{\dot{\tilde{q}}}_{k}^{T}\varphi {\tilde{\theta }}_{k}. \end{array}$$

Substituting $V_{k}\left({\dot{\tilde{q}}}_{k}\left(0\right),{\tilde{q}}_{k}\left(0\right)\right)=0$ and Equation (12) into Equation (8), we have

(13) $$\begin{array}{l} \Delta W_{k}=-V_{k-1}+V_{k}-\frac{1}{2}\int_{0}^{t}\left({\bar{\theta }}_{k}^{T}\Gamma^{-1}{\bar{\theta }}_{k}+2{\bar{\theta }}_{k}^{T}\Gamma^{-1}{\tilde{\theta }}_{k}\right)d\tau \\ \;\leq -V_{k-1}+\int_{0}^{t}{\dot{\tilde{q}}}_{k}^{T}\left(\varphi {\tilde{\theta }}_{k}-K_{D}{\dot{\tilde{q}}}_{k}\right)d\tau \\ \;-\frac{1}{2}\int_{0}^{t}\left({\dot{\tilde{q}}}_{k}^{T}\varphi \Gamma \varphi^{T}{\dot{\tilde{q}}}_{k}+2{\dot{\tilde{q}}}_{k}^{T}\varphi {\tilde{\theta }}_{k}\right)d\tau \\ \;\leq -V_{k-1}-\frac{1}{2}\int_{0}^{t}{\dot{\tilde{q}}}_{k}^{T}\left(\varphi \Gamma \varphi^{T}+2K_{D}\right){\dot{\tilde{q}}}_{k}d\tau \leq 0. \end{array}$$

Through the above proof, it may conclude that *W*_*k*_ is a non-incremental sequence.

b. The proof of the continuity and boundedness of *W*_0_(*t*)

Due to ${\hat{\theta }}_{-1}\left(t\right)=0$, we have ${\hat{\theta }}_{0}\left(t\right)=\Gamma \varphi^{T}\left(q_{0},{\dot{q}}_{0},{\dot{\tilde{q}}}_{0}\right){\dot{\tilde{q}}}_{0}\left(t\right)$, and

(14) $$\begin{array}{l} {Ẇ}_{0}\leq {\dot{\tilde{q}}}_{0}^{T}\left(\varphi \left(q_{0},{\dot{q}}_{0},{\dot{\tilde{q}}}_{0}\right){\tilde{\theta }}_{0}-K_{D}{\dot{\tilde{q}}}_{0}\right)+\frac{1}{2}{\tilde{\theta }}_{0}^{T}\Gamma^{-1}{\tilde{\theta }}_{0}\\ \;\leq -{\dot{\tilde{q}}}_{0}^{T}K_{D}{\dot{\tilde{q}}}_{0}+\left({\hat{\theta }}_{0}^{T}+\frac{1}{2}{\tilde{\theta }}_{0}^{T}\right)\Gamma^{-1}{\tilde{\theta }}_{0}\\ \;=-{\dot{\tilde{q}}}_{0}^{T}K_{D}{\dot{\tilde{q}}}_{0}-\frac{1}{2}{\tilde{\theta }}_{0}^{T}\Gamma^{-1}{\tilde{\theta }}_{0}+\theta^{T}\Gamma^{-1}{\tilde{\theta }}_{0}. \end{array}$$

Due to *a*^2^ + *b*^2^ ≥ 2*ab*, we have $\theta^{T}\Gamma^{-1}\tilde{\theta }\leq K||\Gamma^{-1}{\tilde{\theta }}_{0}|{|}^{2}+\frac{1}{4K}||\theta |{|}^{2}$. The Equation (14) may be rewritten as ${Ẇ}_{0}\leq -\rho_{1}||{\dot{\tilde{q}}}_{0}|{|}^{2}-\rho_{2}||{\tilde{\theta }}_{0}|{|}^{2}+\frac{1}{4K}||\theta |{|}^{2}$, where *K* > 0, ρ_1_ = λ_*min*_(*K*_*D*_), $\rho_{2}=\frac{1}{2}\lambda_{min}\left(\Gamma^{-1}\right)-K\lambda_{max}^{2}\left(\Gamma^{-1}\right)$, and $K\leq \frac{\lambda_{min}\left(\Gamma^{-1}\right)}{2\lambda_{max}^{2}\left(\Gamma^{-1}\right)}$. λ_*min*_(·) and λ_*max*_(·) are respectively the minimum and the maximum eigenvalues of (·). It is obvious that ${Ẇ}_{0}\left(t\right)\leq \frac{\theta_{max}^{2}}{4K}$, that is, *W*_0_(*t*) is continuous and bounded.

c. The proof of continuity and boundedness of *W*_*k*_(*t*)

(15) $$\begin{array}{l} W_{k}=W_{0}+\overset{k}{\underset{j=1}{\sum }}\Delta W_{j}\\ \;\leq W_{0}-\overset{k}{\underset{j=1}{\sum }}V_{j-1}\\ \;\leq W_{0}-\frac{1}{2}\overset{k}{\underset{j=1}{\sum }}{\tilde{q}}_{j-1}^{T}K_{P}{\tilde{q}}_{j-1}-\frac{1}{2}\overset{k}{\underset{j-1}{\sum }}{\dot{\tilde{q}}}_{j-1}^{T}M_{d}{\dot{\tilde{q}}}_{j-1}. \end{array}$$

**Figure 4:**
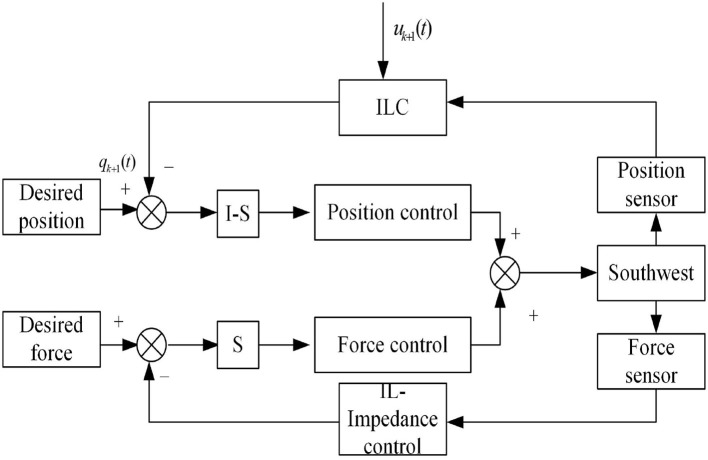
The diagram of iterative learning based impedance control.

Then, $\overset{k}{\underset{j=1}{\sum }}{\dot{\tilde{q}}}_{j-1}^{T}K_{P}{\tilde{q}}_{j-1}+\overset{T}{\underset{j-1}{\sum }}{\dot{\tilde{q}}}_{j-1}^{T}M_{d}{\dot{\tilde{q}}}_{j-1}\leq 2\left(W_{0}-W_{k}\right)\leq 2W_{0}$, and it proves that *W*_*k*_ is bounded. Through the analysis of a, b and c, it can be concluded that $\lim_{k\to \infty }{\tilde{q}}_{k}\left(t\right)=\lim_{k\to \infty }{\dot{\tilde{q}}}_{k}\left(t\right)=0,\forall t\in \left[0,T\right]$. The impedance control combined with the trajectory control form the closed control system of the anthropomorphic arm as shown in Figure 4.

## 4. Numerical Simulations and Real Experiments

Parameters of Southwest in the simulation are set as *m*_1_ = *m*_2_ = 1*kg*, *c*_1_ = *c*_2_ = 0.5*m*, *d*_1_ = *d*_2_ = 0.25*m*, $I_{1}=I_{2}=0.1kg\cdot m^{2}$, *g* = 9.8, *l*_*i*_ = 0.58*m, i* = 1, 2. The position command signals of two joints are respectively sin(2π*t*) and cos(2π*t*). The initial state of the Southwest is set as $\left[q,\dot{q}\right]={\left[0,2\pi ,1,0\right]}^{T}$. Parameters of the impedance controller set as *K*_*p*_ = *K*_*d*_ = *diag*[8, 8] and Γ = *diag*[15, 15, 15, 15, 15].

**Figure 5:**
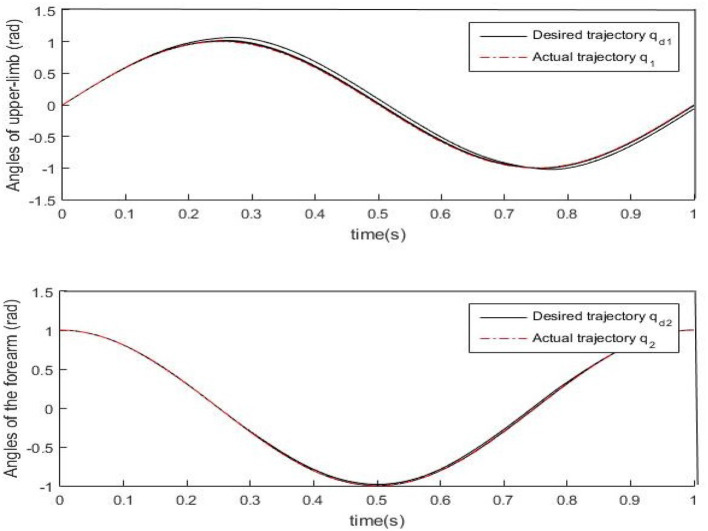
Trajectory errors of the upper-limb and the forearm after 5 iterations.

**Figure 6:**
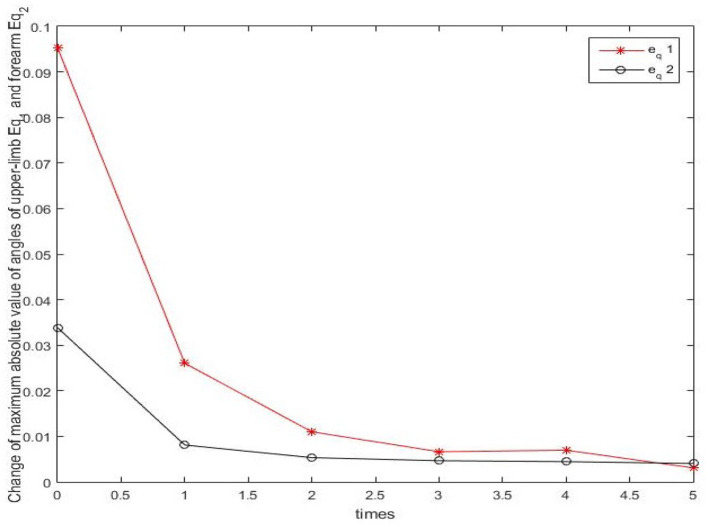
The convergency process of the trajectory tracking in 5 iterations.

**Figure 7:**
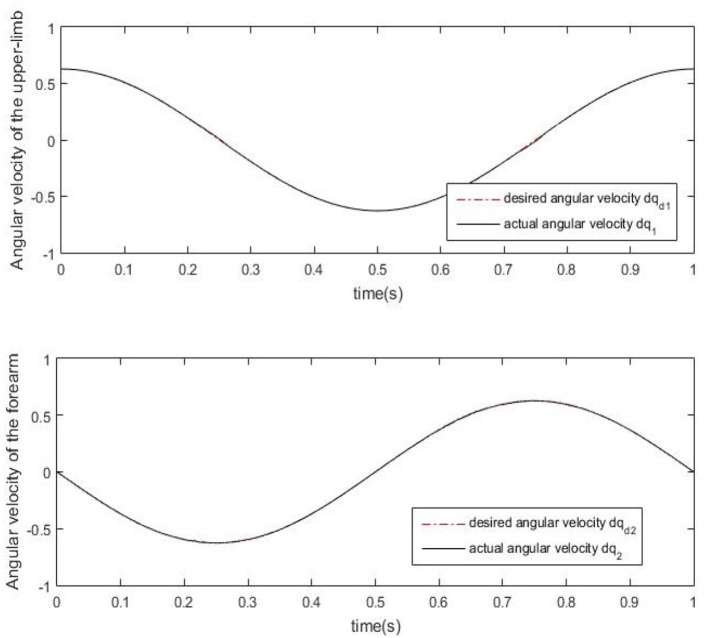
Angular velocity errors of the upper-limb and the forearm after 5 iterations.

**Figure 8:**
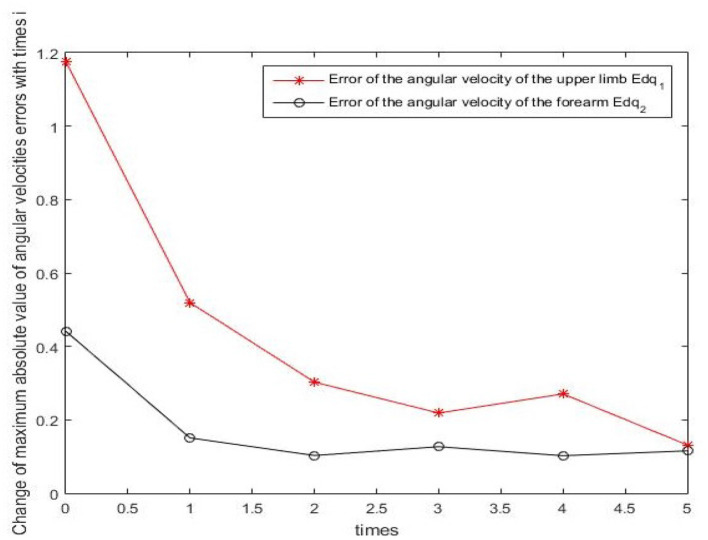
The convergency process of the angular velocity tracking in 5 iterations.

Results of numerical simulations are displayed from Figures 5–8. Angles' errors of the upper-limb and the forearm are illustrated in Figure 5. Desired angles of the upper-limb and the forearm are marked with solid lines while actual angles of the upper-limb and the forearm are marked with dotted lines. The convergent process of the trajectory tracking is showed in Figure 6. Angular velocity errors of the upper-limb and the forearm are illustrated in Figure 7. Desired angular velocity of the upper-limb and the forearm are marked with dotted lines while actual angles of the upper-limb and the forearm are marked with solid lines. The convergent process of the angle tracking is showed in Figure 8. From the results, it can be seen that the process is convergent. After 5 iterations, both of trajectory tracking errors and angular velocity tracking errors are greatly reduced, and the adaptivity of the combined control is improved.

**Figure 9:**
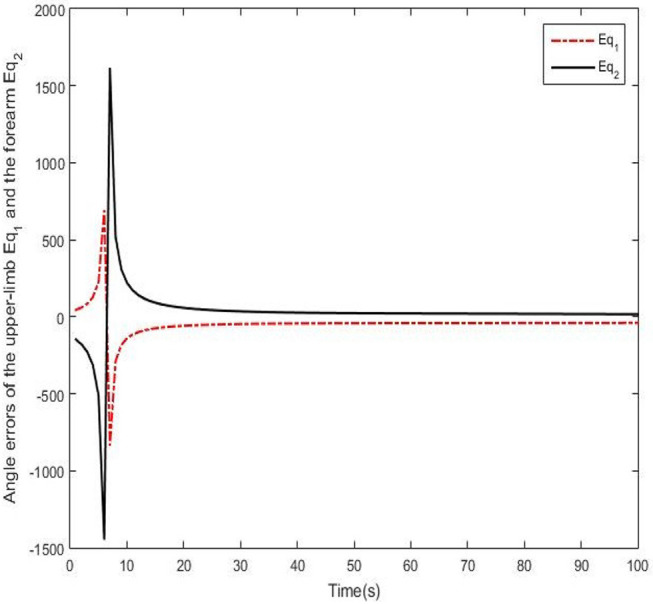
Angle errors of the upper-limb and the forearm in the real experiment.

**Figure 10:**
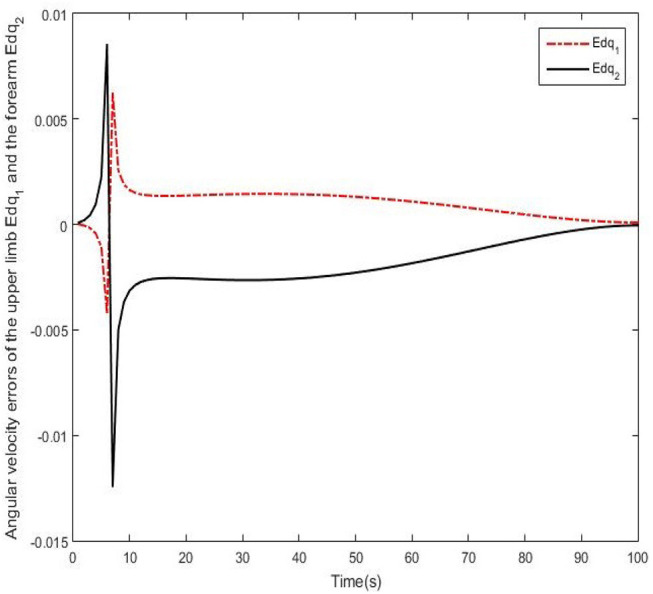
Angular velocity errors of the upper-limb and the forearm in the real experiment.

**Figure 11:**
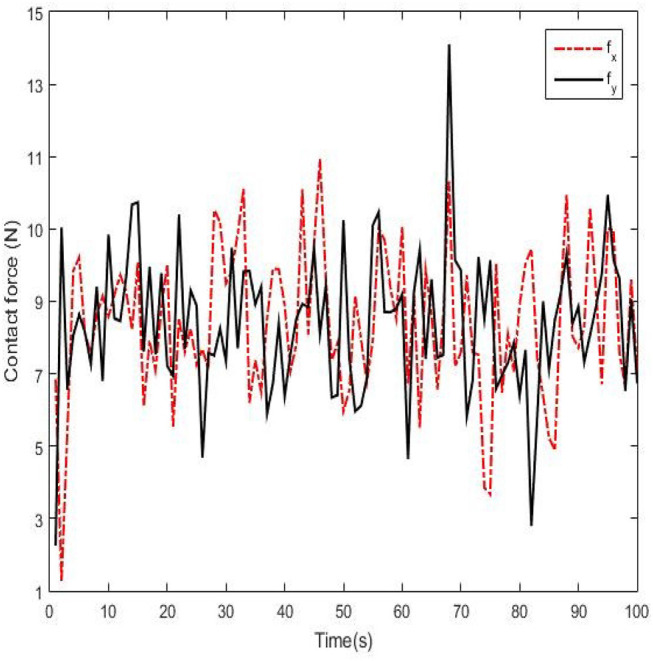
Contact forces of the Southwest anthropomorphic arm along axis-X and axis-Y.

**Figure 12:**
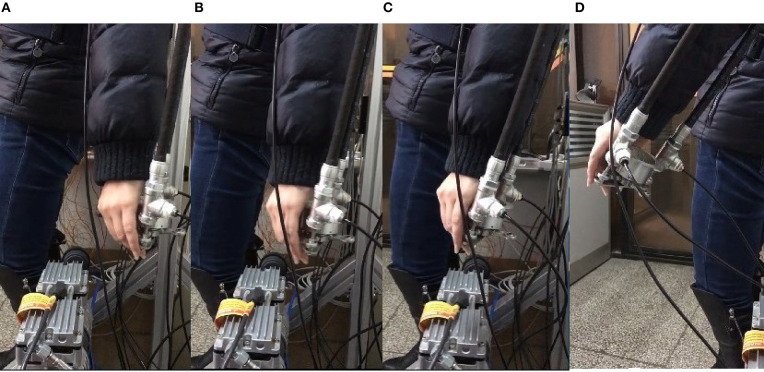
Snapshots of the real experiment. **(A)** Initial state, **(B,C)** Snapshot of the process, **(D)** Final state.

Real experiments are demonstrated from Figures 9–12. Angles' errors of the upper-limb and the forearm in the real experiment are displayed in Figure 9. Angular velocity errors of the upper-limb and the forearm in the real experiment are exhibited in Figure 10. Through the adaptive iterative learning based impedance control, the contact forces along axis-X and axis-Y are illustrated in Figure 11. Figures 12A-D are respectively the initial and final state of the real experiment. Snapshots of the real motion process are showed from Figures 12A-D. From the results, based on the combined control, the Southwest may accurately follow the desired trajectory as well as supply appropriate contact forces to the patient.

From the results of numerical simulations and real experiments, via the proposed control, the end-effecter of the Southeast anthropomorphic arm may acquire appropriate forces to assist impaired upper-limb accomplishing reaching movements. The motion control based on the iterative learning may lead the impaired upper-limb to track the desired trajectory. Simultaneously, the impedance control based on the adaptive iterative learning may adjust the contact force between the end-effecter and the upper-limb so that the upper-limb may well track the trajectory as well as supply the safe contact forces. It can be concluded that the adaptivity of the process is enhanced.

## 5. Conclusion

The paper studies the combined control of the anthropomorphic arm for the purpose of the robot-aided upper-limb rehabilitation therapy exercises. The combined control incorporates the iterative learning motion control and the adaptive iterative based learning impedance control. On one hand, the iterative control aims to control the system accurately follow the desired trajectory through the position sensors. On the other hand, the adaptive iterative learning based control make the system stabilize in the rehabilitation and be compliant to time-varying environment through adjusting contact forces by adaptive parameters of the impedance model. The actual trajectories are adjusted by learning errors and force feedback errors corresponding to the periodic loop and the force loop, so that the actual motion can adapt to the changes of the variant environment. At the same time, the learning database is fine-tuned according to the change of position parameters, which may reduce the influence of disturbances caused by mechanical equipment during the motion. Thus, it minimizes the tracking error and the contact force error, and it may improve the entire force control effect. Through results of numerical simulations and real experiments of the Southwest anthropomorphic arm rehabilitation device, it is proved that the proposed control method has high performances of the robustness and the adaptivity. Future work will focus on applying the proposed control to the real rehabilitation exercises.

## Author Contributions

WT and SA designed the study, performed the research, analyzed data, and wrote the paper.

### Conflict of Interest Statement

The authors declare that the research was conducted in the absence of any commercial or financial relationships that could be construed as a potential conflict of interest.
